# First-Principles Calculations of Hydrogen Solution and Diffusion in 3C-SiC Grain Boundaries

**DOI:** 10.3390/ma18092118

**Published:** 2025-05-05

**Authors:** Yanan Cui, Jingjing Sun, Meng Li, Bingsheng Li

**Affiliations:** 1 Zhengzhou Key Laboratory of Low-Dimensional Quantum Materials and Devices, College of Physics and Optoelectronic Engineering, Zhongyuan University of Technology, Zhengzhou 450007, China; 2022003065@zut.edu.cn (Y.C.); limeng@zut.edu.cn (M.L.); 2 State Key Laboratory of Environmental Friendly Energy Materials, Southwest University of Science and Technology, Mianyang 621010, China; libingshengmvp@163.com

**Keywords:** 3C-SiC, grain boundaries, hydrogen, diffusion

## Abstract

First-principles calculations were employed to study the solution and diffusion properties of hydrogen (H) at the Si-rich and C-rich Σ3(111)[1$\bar{1}$0] ($\Sigma 3_{Si}$ and $\Sigma 3_{C}$) and Σ9(221)[1$\bar{1}$0] (Σ9) grain boundaries (GBs) in 3C-SiC. We constructed GBs of varying sizes and calculated their formation energies and excess volumes to identify the stability of GBs. The Σ9 GB is more stable and has a relatively open structure compared with the Σ3 GB. The solution energies of H at the $\Sigma 3_{Si}$, $\Sigma 3_{C}$ and Σ9 GBs are significantly reduced to 1.46, 2.30 and 1.47 eV, respectively. These values are much lower than that in the bulk. The negative segregation energies indicate that H is more likely to reside at the GBs rather than in the bulk. The diffusion energy barrier of H in the $\Sigma 3_{C}$ GB is as high as 1.27 eV, whereas in the $\Sigma 3_{Si}$ GB and Σ9 GB, the barriers are as low as 0.42 eV and 0.28 eV, respectively. These results suggest that H migration will be suppressed in the $\Sigma 3_{C}$ GB but promoted in the $\Sigma 3_{Si}$ and Σ9 GBs. The differences in H diffusion behavior among these three GBs may be attributed to the relatively more open structures of the $\Sigma 3_{Si}$ and Σ9 GBs compared with the $\Sigma 3_{C}$ GB. These results are essential for understanding the diffusion mechanism of H and its retention behavior in SiC.

## 1. Introduction

Silicon carbide (SiC) ceramic materials exhibit many excellent properties, including high breakdown voltage, a wide bandgap, and resistance to high temperature, high pressure, corrosion, and radiation damage. Due to their superior properties, 4H-SiC and 6H-SiC are widely utilized in high-power electronic devices [1], while 3C-SiC and SiC composites—predominantly consisting of 3C-SiC—are regarded as promising candidates for structural materials in high-temperature nuclear fission reactors and future fusion reactors [2,3]. In high-temperature gas-cooled reactors, SiC is used as the fission products’ barrier for three-layer-coated isotropic spherical fuel particles [4]. For nuclear fusion applications, incorporating a thin SiC layer on tungsten or eurofer is expected to enhance the control of the tritium inventory [5,6,7]. Additionally, using SiC inserts in tritium blankets should improve corrosion resistance [7]. In these applications, SiC is radiated by high-energy particles in the reactors, including high-energy neutrons and hydrogen (H) isotopes, which produce a large number of point defects in its interior and induce amorphization in SiC [8]. Furthermore, transmutation results in the formation of solid elements such as magnesium and boron, as well as gaseous elements including H [9,10]. Irradiation-induced defects and grain boundaries (GBs) are generally the trapping source for H in solids [11,12]. H exhibits a series of behaviors inside SiC, such as diffusion, clusters, and interactions with various defects, particularly GBs [13,14]. These behaviors lead to the formation of H bubbles and volume expansion in SiC, thereby affecting material properties and reducing service life [15]. On the other hand, the diffusion of the H isotope tritium in SiC can also affect the self-sustaining tritium inventory and tritium safety in fusion reactors [16].

The experimental and simulation results of past studies show that GBs play a crucial role in the behaviors of impurities in SiC [17,18,19,20]. The influence of different GBs in SiC on the diffusion behavior of impurity atoms is distinct, which may be related to the GB structures [21,22,23]. The coincident site lattice (CSL)-related GBs (e.g., $\Sigma 3^{n}$) occupy the majority of GBs in SiC [24,25]. So far, most studies have focused on the behavior of H in SiC bulks, but the interaction mechanism between H and SiC GB is still unclear. The diffusion barrier of H in 3C-SiC bulk was calculated to be 0.5 eV by density functional theory, and the diffusion barriers of H in the presence of silicon (Si) and carbon (C) vacancies are, respectively, as high as 1.09 and 1.71 eV [26,27]. Esteban et al. and Tam et al. measured the diffusion energy barriers of deuterium and tritium in SiC by experimental observations, obtaining values of about 1.12 eV [16,28]. However, the tritium diffusion energy barrier was reported to be 2.82 eV by Causey et al. [29]. It can be found that the values for H exhibit large discrepancies. The difference may result from the different research techniques and the trapping effect of defects for H in SiC. Various defects, including vacancies, interstitial atoms, and GBs, can significantly influence H diffusion within SiC. Among these, GBs represent important intrinsic defects in SiC and may play a crucial role in facilitating the diffusion of H atoms and the formation of H bubbles. Therefore, studying the solution and diffusion behaviors of H in 3C-SiC GBs can help us better understand the experimental results and the material properties.

In this paper, first-principles calculations were used to study the diffusion behaviors of H at the three GBs: Si-rich and C-rich Σ3(111)[1$\bar{1}$0], and Σ9(221)[1$\bar{1}$0] in 3C-SiC. Firstly, we constructed the structures of the three GBs with different C-Si layers and obtained the GB energy. The influence of different C-Si layers on the H solution was investigated in the GB supercells. Then, the solution and segregation energies of H at the GBs were studied. Finally, the diffusion energy barriers of H along the GBs were calculated to further understand the diffusion mechanism.

## 2. Computational Details

The first-principles calculations based on density functional theory were performed using the Vienna ab initio simulation software package (VASP) [30,31]. The interaction between ions and electrons was expressed by the projected augmented wave potential (PAW) method. The exchange correlation function was described with the plane wave method (PBE) in generalized gradient approximation (GGA) [32,33]. The maximum number of steps of ion relaxation in the calculation was 300. The plane wave truncation energy was 520 eV, and the convergence force on each atom was 0.01 eV/Å. The equilibrium lattice constant for 3C-SiC was optimized to be 4.38 Å. The diffusion properties were calculated using the climbing image nudged elastic band method (CL-NEB) [34]. The thermodynamic stability of GBs can be evaluated by the GB energy $\gamma_{GB}$, which is the excess energy of a system containing GB per unit area [35]. The calculation formula for $\gamma_{GB}$ is

(1) $$\gamma_{GB}=\frac{E_{GB}-NE_{SiC}}{2S}$$

where $E_{GB}$ is the total energy of the GB supercell, and $E_{SiC}$ is the total energy per formula unit of 3C-SiC. *N* is the number of SiC formula units appearing in the GB supercell, and *S* is the cross section area of GB plane. In addition, GB characteristic is always associated with excess volume Δ*V*. Δ*V* is the excessive volume of a system containing GB per unit area [36]. Δ*V* is calculated by

(2) $$\Delta V=\frac{V_{GB}-N*V_{SiC}}{2S}$$

where $V_{GB}$ is the volume of the GB supercell, and $V_{SiC}$ is the volume of C–Si atomic pair in the bulk. The solution energy $E_{H}^{sol}$ is the energy required to embed a H atom into SiC bulk or GB, which is given as

(3) $$E_{H}^{sol}=E_{GB/Bulk+H}-E_{GB/Bulk}-\frac{1}{2}H_{2}$$

where $E_{GB/Bulk+H}$ represents the total energy of the GB supercell or the 3C-SiC lattice with an interstitial H, $E_{GB/Bulk}$ represents the total energy of the GB supercell or the 3C-SiC lattice without H, and $H_{2}$ refers to the energy of a H molecule. The calculation formula for the segregation energy $E_{H}^{Seg}$ of H at the GB site is as follows:

(4) $$E_{H}^{Seg}=E_{GB+H}^{sol}-E_{Bulk+H}^{sol}$$

where $E_{GB+H}^{sol}$ represents the total energy of the GB supercell containing H, and $E_{Bulk+H}^{sol}$ represents the solution energy of the H at the most stable site in the perfect SiC bulk.

## 3. Results and Discussion

### 3.1. Hydrogen in Perfect 3C-SiC

**Figure 1:**
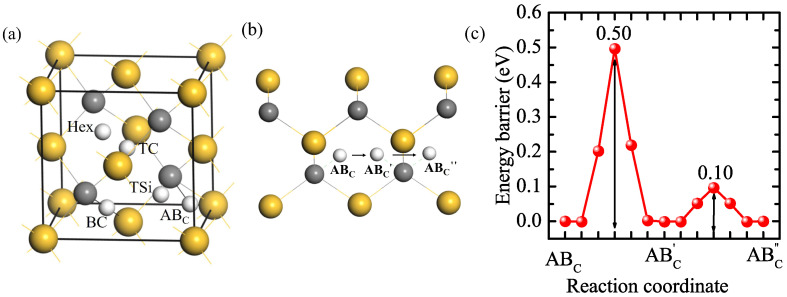
The five initial sites of H in SiC bulk (**a**), the diffusion path (**b**), and diffusion energy barrier (**c**) of H in bulk 3C-SiC. Gray, yellow, and white spheres represent C, Si, and H atoms, respectively.

The solution and diffusion behaviors of H in a perfect 3C-SiC bulk were investigated as a reference. The supercell of 3C-SiC contains 108 C and 108 Si atoms. There are five initial interstitial sites: the tetrahedral center site of four Si atoms (TSi), the tetrahedral center site of four C atoms (TC), the hexagonal site neighboring three C and three Si atoms (Hex), the bond center site of C and Si atoms (BC), and the site along the anti-bonding direction of C-Si bonds (AB_*C*_) [37,38]. H at the Hex site is not stable, which moves to the TSi site. The calculated solution energies for H at the other four stable interstitial sites are shown in Table 1. The solution energies for H at the TC, TSi, and BC sites are 3.61, 2.93, and 2.60 eV, respectively, much higher than that of H at the AB_*C*_ site (2.48 eV). Hence, the solution energies follow the following order: TC > TSi > BC > AB_*C*_, where AB_*C*_ is the most stable interstitial site for H. Our results are consistent with the previous calculation results [39,40,41]. The diffusion path of H in the perfect 3C-SiC bulk and the corresponding diffusion energy barriers of H are shown in Figure 1b,c. The energy barrier for a H atom jumping from an AB_*C*_ position on the C atom to the equivalent position AB′_*C*_ on the nearest neighboring C atom is 0.50 eV. The H can also rotate around a C atom between AB′_*C*_ and AB″_*C*_, and the barrier is just 0.10 eV. It can be known that the migration energy barrier of H in the perfect 3C-SiC bulk is 0.50 eV, the same as the result of Kaukonen et al. [27].

### 3.2. The Structures of Different Grain Boundaries

**Figure 2:**
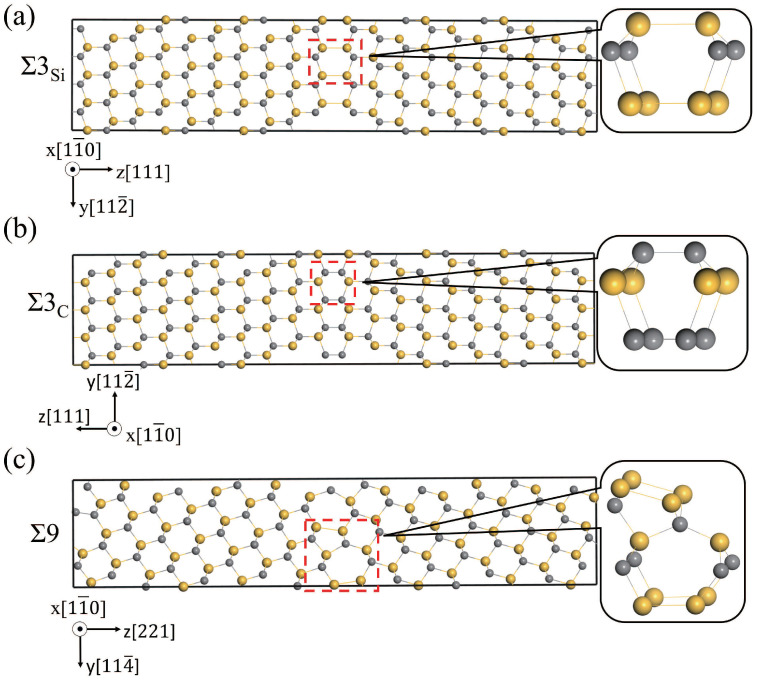
Structures of three GBs, $\Sigma 3_{Si}$ (**a**), $\Sigma 3_{C}$ (**b**), and Σ9 (**c**), projected in the $\left[1\bar{1}0\right]$ direction. Gray and yellow spheres are used to represent C and Si atoms, respectively.

Three GBs, i.e., Si-rich and C-rich Σ3(111)[1$\bar{1}$0] ($\Sigma 3_{Si}$ and $\Sigma 3_{C}$) and Σ9(221)[1$\bar{1}$0] (Σ9), were studied and discussed, and were constructed using the coincidence site lattice model. The structures of $\Sigma 3_{Si}$, $\Sigma 3_{C}$, Σ9, and the basic structural units in the GBs are shown in Figure 2. The supercells of $\Sigma 3_{Si}$ and $\Sigma 3_{C}$ contain six C-Si atomic layers, and fifteen C-Si atomic layers are in the Σ9 supercell. The structural units of $\Sigma 3_{Si}$ and $\Sigma 3_{C}$ GBs have a high similarity, all of which contain six atom rings [42,43]. Σ9 GB is composed of two different polyhedrons with seven-atom rings and five-atom rings, respectively. The excess volumes of the GBs were calculated to be 0.27, 0.07, and 0.07 Å for Σ9, $\Sigma 3_{C}$, and $\Sigma 3_{Si}$, respectively. The excess volume of Σ9 is much higher than that of Σ3, indicating that Σ9 has a much more open structure than Σ3. Since both the supercells of $\Sigma 3_{Si}$ and $\Sigma 3_{C}$ contain a Si-rich GB and a C-rich GB, the excess volumes are the same. The Si-Si bond length in the Si-rich GB is 2.40 Å, and the C-C bond length in the C-rich GB is 1.64 Å. The Si-rich GB is more open than the C-rich GB.

**Figure 3:**
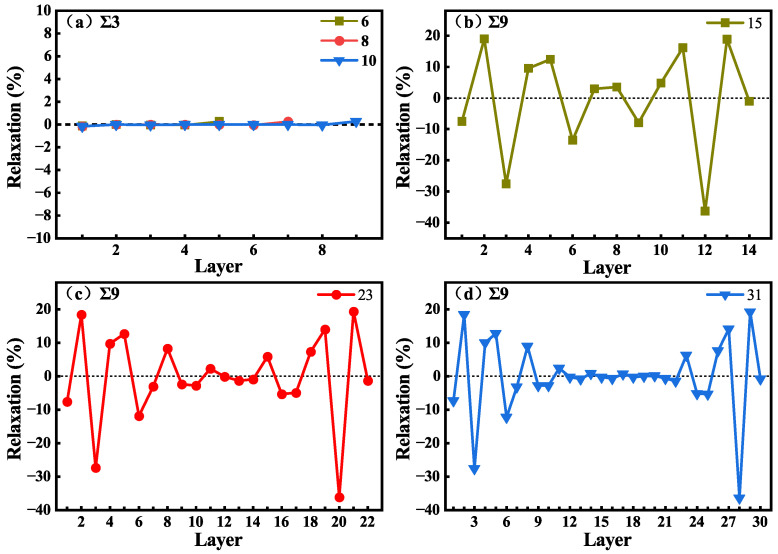
Changes in layer spacing as a function of the number of layers away from the $\Sigma 3_{C}$ GB with 6, 8, and 10 C-Si atomic layers (**a**), and Σ9 GBs with 15 (**b**), 23 (**c**), and 31 (**d**) C-Si atomic layers.

As shown in Figure 2, no vacuum layer was added when constructing the GB structures. To reduce the effects of interactions between periodic images in subsequent calculations, the GB size should be large enough to eliminate interactions between the two GBs, and the system should contain a bulk-like environment in the supercell. The structures of GBs with different C-Si atomic layers were constructed, and the GB energies were calculated and are shown in Table 2. The parameters of $\Sigma 3_{Si}$ and $\Sigma 3_{C}$ GBs are the same, because their supercells are essentially the same and the $\gamma_{GB}$ is the average energy of a Si-rich GB and a C-rich GB in the supercell. The $\gamma_{GB}$ of $\Sigma 3_{C}$ and $\Sigma 3_{Si}$ GB with 6, 8, and 10 C-Si atomic layers are 1.39 J/m^2^. The $\gamma_{GB}$ of Σ9 GB with 15, 23, and 31 C-Si atomic layers are 1.35 J/m^2^. The values of $\gamma_{GB}$ for the GB systems agree well with the previous calculation results [17]. In addition, for Σ9 GB, a 3 × 1 × 1 supercell containing 23 C-Si atomic layers was calculated, and other supercells are 2 × 1 × 1, which verifies that in the $\left[1\bar{1}0\right]$ direction, the periodic interaction has no effect on the results. As shown in Table 2, it can be found that the $\gamma_{GB}$ of the three GBs does not change with the increase in atomic layer number. The results suggest that GB sizes are large enough to eliminate interactions between the two GBs in the supercell. To verify that the GBs contain a bulk-like structure, the changes in C-Si atomic layer spacing ($\Delta_{ij}$) in the supercell were calculated. $\Delta_{ij}$ is defined as the percentage change in the vertical positions of two subsequent atomic layers (d_*i*_ and d_*j*_) with respect to the inter-planar distance (d) in the bulk system, which can be written as $\Delta_{ij}$ = [(d_*j*_ − d_*i*_) − d]/d. The changes in atomic layer spacing in the [111] direction for $\Sigma 3_{C}$ GB and in the [221] direction for Σ9 GB are presented in Figure 3 as a function of the layer away from the grain boundary plane. In the other two directions, the changes are neglected. Figure 3a displays the changes in the layer spacing of $\Sigma 3_{C}$ GBs with 6, 8, and 10 C-Si atomic layers. It can be seen that the changes are close to zero, revealing very small relaxations between the atomic layers. However, in Figure 3b–d, the spacing changes of Σ9 GB with 15, 23, and 31 C-Si atomic layers are different. It can be found that the change in layer spacing near the GB fluctuates greatly and shows a trend of oscillation. The expansion of $\Delta_{23}$ is up to about 18%, and the contraction of $\Delta_{34}$ is up to about 28%. In the 31 atomic layers of the Σ9 GB, the negligible changes for the layers from 12 to 22 indicate that the atomic relaxation is localized only within 12 layers from the GB. Therefore, the supercell containing 31 layers between the two GBs is large enough to reflect the characteristics of the GB and provides a bulk-like area.

### 3.3. The Solution Energy of Hydrogen near the Grain Boundaries

**Figure 4:**
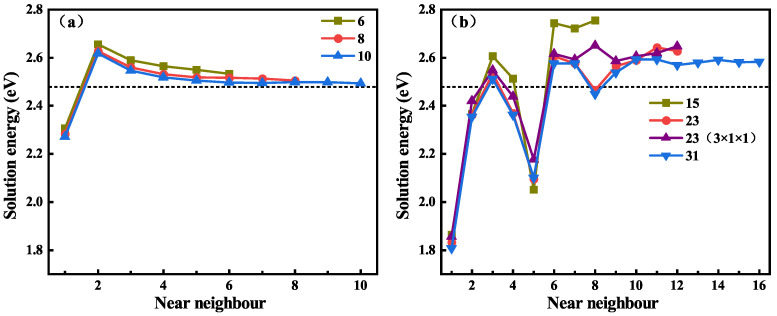
The solution energies of H at different AB_*C*_ sites near the $\Sigma 3_{C}$ GBs with 6, 8, and 10 C-Si atomic layers (**a**) and Σ9 GBs with 15, 23, and 31 C-Si atomic layers (**b**), respectively.

The solution behavior of H at the AB_*C*_ site on different C-Si layers near the GB in the supercell was calculated, and the solution energy for H at different neighboring locations along the GB is shown in Figure 4, where the black dotted line represents the lowest solution energy of H in the perfect system. Figure 4a is the solution energy of H near the $\Sigma 3_{C}$ GB, and the $\Sigma 3_{C}$ GBs have three different C-Si atomic layers. It can be seen that the three curves of the solution energies show the same trend. The solution energy of H near the 6-layer $\Sigma 3_{C}$ GB is the largest, and the solution energy of H near the 8-layer and 10-layer $\Sigma 3_{C}$ GBs are very close, but only the curve of the solution energies along the 10-layer $\Sigma 3_{C}$ GB shows convergence. Combined with the content of the previous section, it was decided to use 10-layer $\Sigma 3_{C}$ and $\Sigma 3_{Si}$ GBs for subsequent research. Figure 4b shows the solution energy of H in the vicinity of Σ9 GB, and the Σ9 GB contains three different C-Si atomic layers and a 3 × 1 × 1 structure of 23 atomic layers. For the 15, 23, and 31-layer GBs, the solution energies of H on the nearest 8, 12, and 16 layers were calculated, respectively. It can be seen that the variation trend and the value of the solution energy of H on the first six layers of the four GBs are almost similar, and the differences become relatively large thereafter. The solution energy begins to converge steadily from the sites of the tenth atomic layer. By comparing the solution energy curves of H near the two different 23-layer GBs, it can be found that they are very close to each other. Hence, the thickness change in the $\left[1\bar{1}0\right]$ direction has little effect on the results, and the 2 × 1 × 1 supercell of the 31-layer GB was used for subsequent calculations. In addition, the influence range of Σ9 GB on the solution energy of H is greater than that of the $\Sigma 3_{C}$ GB. This is attributed to the more open structure of the Σ9 GB compared with the $\Sigma 3_{C}$ GB [44]. Overall, the solution energy of H near the GBs is significantly lower than that of H in the bulk-like regions. This suggests that GBs may be a deep trapping region for H atoms.

### 3.4. The Solution and Segregation of Hydrogen at Grain Boundaries

**Figure 5:**
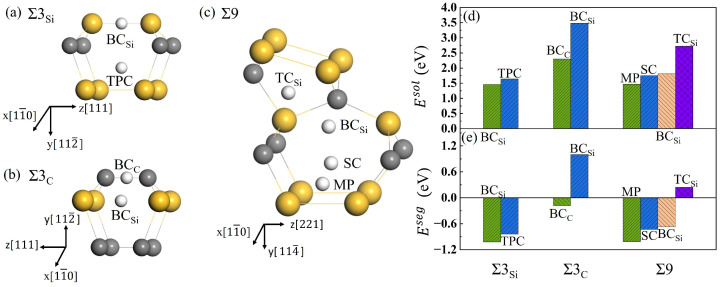
The interstitial sites for H at $\Sigma 3_{Si}$ GB (**a**), $\Sigma 3_{C}$ GB (**b**), and Σ9 GB (**c**), and the solution energies (**d**) and segregation energies (**e**) of H in different interstitial positions along the three GBs. ${\mathrm{BC}}_{Si}$ is the position of the Si-Si bond center, and ${\mathrm{BC}}_{C}$ is the center of the C-C bond. TPC is the site of the triangular prism center, ${\mathrm{TC}}_{Si}$ is the tetrahedral center of four Si atoms, SC is the center of six silicon atoms, and MP is the position in the mid-vertical plane of the Si-Si bond. Gray, yellow, and white spheres represent C, Si, and H atoms, respectively.

To explore the diffusion characteristics of H in the GB of SiC, it is necessary to understand its solution and segregation properties. $\Sigma 3_{C}$ and $\Sigma 3_{Si}$ GBs have similar structures and high symmetry, and the initial interstitial sites for H were selected as similar high symmetry ones. In the Σ9 GB, eleven possible initial sites were investigated. After structural optimization, the stable positions for H in the three GBs are shown in Figure 5a–c. The solution energies and segregation energies for H were calculated using Equations (3) and (4), and shown in Figure 5d,e, respectively. For the $\Sigma 3_{Si}$ GB, the two stable interstitial sites for H are the Si-Si bond center (${\mathrm{BC}}_{Si}$) and the triangular prism center (TPC) composed of six Si atoms. The solution energies for H in the position of ${\mathrm{BC}}_{Si}$ and TPC are 1.46 and 1.69 eV, and the segregation energies are −1.02 eV and −0.79 eV, respectively. Hence, the lowest-energy interstitial sites for H at $\Sigma 3_{Si}$ GB is ${\mathrm{BC}}_{Si}$. For the $\Sigma 3_{C}$ GB, the two interstitial sites are the center of the C-C bond (${\mathrm{BC}}_{C}$) and ${\mathrm{BC}}_{Si}$. The solution energy for H at the ${\mathrm{BC}}_{C}$ site is 2.30 eV, much lower than that for H at the ${\mathrm{BC}}_{Si}$ site (3.48 eV). The corresponding segregation energies are −0.18 eV and 1.00 eV, respectively. This indicates that H prefers the ${\mathrm{BC}}_{C}$ site rather than the ${\mathrm{BC}}_{Si}$ site. There are four interstitial sites for H at the Σ9 GB. They are the position in the mid-vertical plane of the Si-Si bond (MP), the center of six Si atoms (SC), the tetrahedral center of four Si atoms (${\mathrm{TC}}_{Si}$), and ${\mathrm{BC}}_{Si}$. The solution energies for H are 1.47, 1.75, 2.72, and 1.81 eV at the four sites, respectively, and the segregation energies are, respectively, −1.01, −0.73, 0.24, and −0.67 eV. The solution energy for H at MP is much lower than those at the other three sites. MP is the most stable site for H at Σ9. The solution energies of H in $\Sigma 3_{Si}$ and Σ9 are much lower than that in $\Sigma 3_{C}$, which may due to the more open structure of $\Sigma 3_{Si}$ and Σ9 compared with $\Sigma 3_{C}$. Additionally, the solution energies of H in the three GBs are much lower than that in the perfect SiC bulk, and the segregation energies are negative. The results indicate that H is more likely to segregate in the three GBs rather than in the bulk. Our results here are in agreement with that of Meng et al. [18].

**Figure 6:**
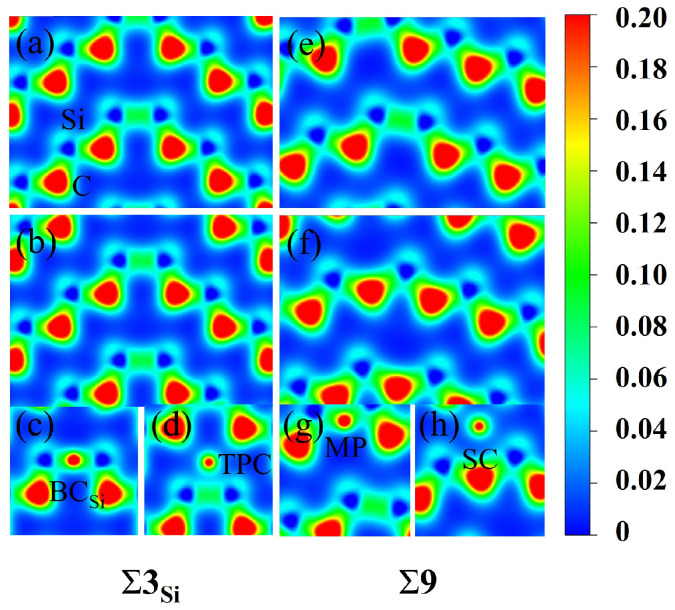
The charge density maps of [1$\bar{1}$0] planes in the $\Sigma 3_{Si}$ and Σ9 supercells. (**a**,**b**) are the two different atomic layers without H in the [1$\bar{1}$0] direction of $\Sigma 3_{Si}$, while (**e**,**f**) are the two different atomic layers free of H in the [1$\bar{1}$0] direction of Σ9. The charge density maps of H at the ${\mathrm{BC}}_{Si}$ (**c**), TPC (**d**) sites in $\Sigma 3_{Si}$ GB and the MP (**g**) and SC (**h**) sites in Σ9 GB are shown. The unit is e/bohr^3^.

**Figure 7:**
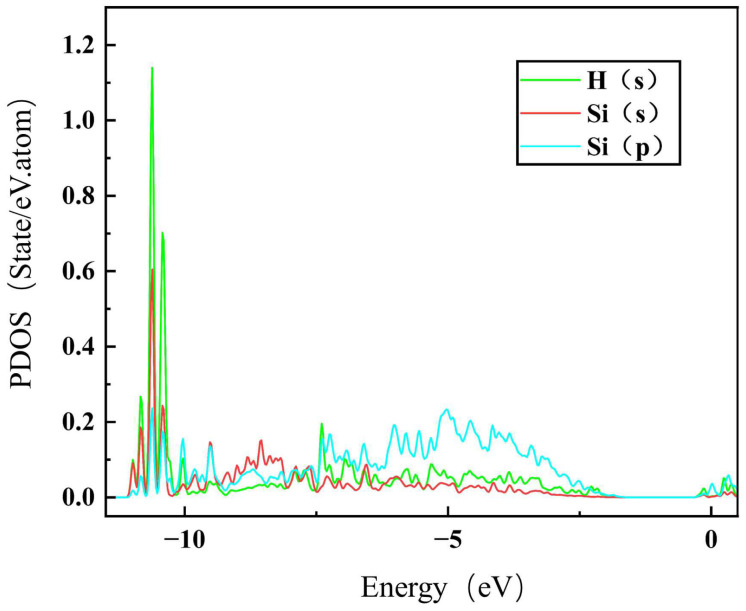
The PDOS for the hybridization between Si and H at the MP site of Σ9.

To further understand the segregation behavior of H in SiC GBs, the charge density of the above different configurations were calculated here [45]. The charge density maps of $\Sigma 3_{Si}$ and Σ9 GBs without H are shown in Figure 6, where a, b and e, f represent the charge density maps of two different atomic layers without interstitial H in the $\left[1\bar{1}0\right]$ direction. The charge densities of H at the sites ${\mathrm{BC}}_{Si}$ and TPC in the $\Sigma 3_{Si}$ GB, and MP and SC in the Σ9 GB are also presented in Figure 6 for comparison. The charge density between H and Si atoms in the ${\mathrm{BC}}_{Si}$ and MP configurations is denser than that in the TPC and SC configurations in $\Sigma 3_{Si}$ and Σ9 GBs, respectively. H at MP and ${\mathrm{BC}}_{Si}$ sites has a stronger bond with its surrounding Si atoms, while H at TPC and SC sites are in a relatively isolated state and has a weak interaction with its surrounding atoms. Figure 7 shows the partial density of states (PDOSs) of H in the MP position of the Σ9 GB. The s orbital of H and the s and p orbitals of the closest Si are shown in the figure. It can be found that the s orbitals of H and Si have a hybridization peak near −11 eV, which indicates that there is a chemical bond formation between them. Therefore, the reason for the low solution energy of H at the MP and ${\mathrm{BC}}_{Si}$ sites may be that the H atom is more active and easy to form chemical bonds with its surrounding atoms, which is similar to the behavior of oxygen, magnesium, and other elements in SiC GBs [17,18].

### 3.5. The Diffusion of Hydrogen in Grain Boundaries

**Figure 8:**
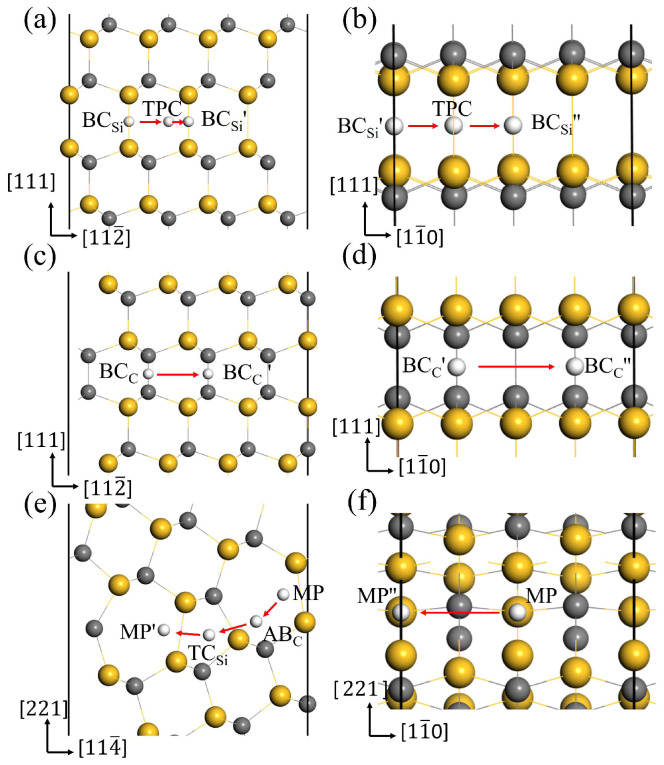
The optimal diffusion paths for H in the directions $\left[11\bar{2}\right]$ (**a**), $\left[1\bar{1}0\right]$ (**b**) of $\Sigma 3_{Si}$ GB, $\left[11\bar{2}\right]$ (**c**), $\left[1\bar{1}0\right]$ (**d**) of $\Sigma 3_{C}$ GB, $\left[11\bar{4}\right]$ (**e**), $\left[1\bar{1}0\right]$ (**f**) of Σ9 GB.

**Figure 9:**
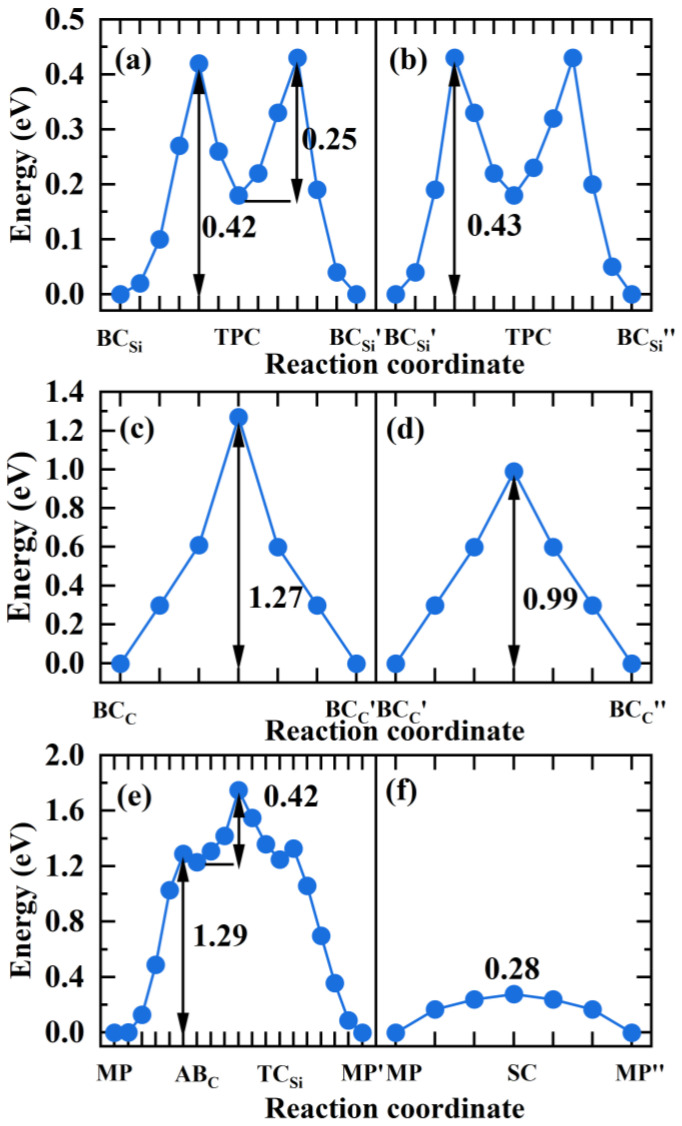
The diffusion energy profile of H atom migrating in the directions $\left[11\bar{2}\right]$ (**a**), $\left[1\bar{1}0\right]$ (**b**) of $\Sigma 3_{Si}$ GB, $\left[11\bar{2}\right]$ (**c**), $\left[1\bar{1}0\right]$ (**d**) of $\Sigma 3_{C}$ GB, $\left[11\bar{4}\right]$ (**e**), $\left[1\bar{1}0\right]$ (**f**) of Σ9 GB.

In the above section, the most stable interstitial sites for H in GBs have been obtained. The diffusion behavior of H along the 3C-SiC GBs was investigated using the CL-NEB method, and the optimal diffusion paths for H in the two directions of GBs were obtained. Figure 8 displays the diffusion paths of H along the two directions at the GB interface, and the corresponding migration energy barriers are shown in Figure 9.

For the $\Sigma 3_{Si}$ GB, ${\mathrm{BC}}_{Si}$ is the most stable site for H. ${\mathrm{BC}}_{Si}^{\prime }$ and ${\mathrm{BC}}_{Si}^{\prime \prime }$ in Figure 8a,b are the equivalent sites of ${\mathrm{BC}}_{Si}$. In the $\left[11\bar{2}\right]$ and $\left[1\bar{1}0\right]$ directions, H prefers to migrate between the two most stable sites through the metastable site TPC. In the path ${\mathrm{BC}}_{Si}$→TPC→${\mathrm{BC}}_{Si}^{\prime }$, H needs to overcome the energy barrier of 0.42 eV to reach the TPC site, and then overcome the energy barrier of 0.25 eV to reach the ${\mathrm{BC}}_{Si}^{\prime }$ site in Figure 9a. Therefore, the diffusion barrier of H in the $\left[11\bar{2}\right]$ direction of $\Sigma 3_{Si}$ GB is 0.42 eV. In the $\left[1\bar{1}0\right]$ direction, the diffusion path for H atom is ${\mathrm{BC}}_{Si}^{\prime }$→TPC→${\mathrm{BC}}_{Si}^{\prime \prime }$, and the energy barrier is 0.43 eV in Figure 9b. Hence, the diffusion activation energies of H in the two directions of $\Sigma 3_{Si}$ GB are lower than that in the bulk region (0.50 eV), indicating that the $\Sigma 3_{Si}$ GB may accelerate H migrating in 3C-SiC. When H diffuses along the $\Sigma 3_{C}$ GB, the migration paths for H along the $\left[11\bar{2}\right]$ and $\left[1\bar{1}0\right]$ directions are ${\mathrm{BC}}_{C}$→${\mathrm{BC}}_{C}^{\prime }$ and ${\mathrm{BC}}_{C}^{\prime }$→${\mathrm{BC}}_{C}^{\prime \prime }$ in Figure 8c,d, respectively. The activation energy barrier for H migrating between the ${\mathrm{BC}}_{C}$ and ${\mathrm{BC}}_{C}^{\prime }$ sites is as high as 1.27 eV in Figure 9c, and H needs to overcome the energy barrier of 0.99 eV to reach the ${\mathrm{BC}}_{C}^{\prime \prime }$ position from the ${\mathrm{BC}}_{C}^{\prime }$ site in Figure 9d. The diffusion energy barriers of H in the two directions along the $\Sigma 3_{C}$ GB are much larger than that of H in perfect SiC bulk. This suggests that H diffusion in the $\Sigma 3_{C}$ GB plane is more difficult than in the bulk.

For the Σ9 GB, we investigated H migrating between the most stable interstitial sites MP and its equivalent sites ${\mathrm{MP}}^{\prime }$ and ${\mathrm{MP}}^{\prime \prime }$. The diffusion path for H along the $\left[11\bar{4}\right]$ direction is MP→AB_*C*_→${\mathrm{TC}}_{Si}$→${\mathrm{MP}}^{\prime }$, as shown in Figure 8e. Starting from the MP site, H needs to overcome the energy barrier of 1.29 eV to reach the AB_*C*_ site, then it must surmount the energy barrier of 0.42 eV to reach the ${\mathrm{TC}}_{Si}$ site in the 5-atom ring, ultimately reaching the stable site of ${\mathrm{MP}}^{\prime }$. Therefore, the diffusion barrier of H in the $\left[11\bar{4}\right]$ direction of Σ9 GB is 1.29 eV, much larger than 0.5 eV. The diffusion path for H in the $\left[1\bar{1}0\right]$ direction is shown in Figure 8f, and the diffusion energy barrier is shown in Figure 9f. H hops between MP to ${\mathrm{MP}}^{\prime \prime }$ and proceeds via the metastable site SC. The diffusion barrier is 0.28 eV, much lower than 0.5 eV. The results show that the diffusion of H in the two directions of Σ9 GB is anisotropic. The diffusion of H along the $\left[11\bar{4}\right]$ direction is difficult, while the diffusion of H in the $\left[1\bar{1}0\right]$ direction is easy. The MP→${\mathrm{MP}}^{\prime \prime }$ along the $\left[1\bar{1}0\right]$ direction of the Σ9 GB is likely to serve as a fast diffusion path for H. This is the same as the diffusion behavior of helium in the Σ9 GB of 3C-SiC [46]. This phenomenon may be attributed to the diffusion path of H within an open structure containing seven-atom rings along the $\left[1\bar{1}0\right]$ direction. While H diffuses along the $\left[11\bar{4}\right]$ direction, it needs to pass the region with high atomic density, so the diffusion energy barrier is relatively high. This is similar to the diffusion phenomenon of H in the Σ9 GB of γ-Fe obtained by He et al. [47].

## 4. Conclusions

The solution and diffusion properties of H in the three grain boundaries (GBs), Si-rich and C-rich Σ3(111)[1$\bar{1}$0] ($\Sigma 3_{Si}$ and $\Sigma 3_{C}$) and Σ9(221)[1$\bar{1}$0] (Σ9) of 3C-SiC, were studied using first-principles calculations. The dimensions of the constructed GB supercells are sufficiently large to ensure the accuracy of the investigations. The GB energy of Σ9 was found to be 1.35 eV, slightly smaller than that of Σ3 (1.39 eV). The solution energies of H in the region near the GBs are significantly lower than that in the bulk. The negative segregation energies indicate that H is more likely to segregate in the three GBs rather than in the bulk. H in the GBs exhibits a greater tendency to form stable chemical bonds with its nearest neighboring atoms. The diffusion energy barrier of H in the $\Sigma 3_{C}$ GB is as high as 1.27 eV, whereas in the $\Sigma 3_{Si}$ GB and Σ9 GB, the barriers are as low as 0.42 and 0.28 eV, respectively. These results suggest that the migration of H in the $\Sigma 3_{C}$ GB will be suppressed, but promoted in the $\Sigma 3_{Si}$ GB and Σ9 GB. The differences in H diffusion behavior among these three GBs may be attributed to the relatively more open structures of the $\Sigma 3_{Si}$ and Σ9 GBs compared with the $\Sigma 3_{C}$ GB. Our theoretical findings provide foundational data for understanding the diffusion mechanism of H and its retention behavior at GBs in SiC.

## Figures and Tables

**Table 1:** The solution energies of H at five different interstitial sites in 3C-SiC bulk. Units are eV.

Site	TC	TSi	Hex	BC	AB_*C*_
This work	3.61	2.93	TSi	2.60	2.48
Ref. [39]	3.05	2.61	TSi	2.41	2.05
Ref. [40]	3.36	2.93	TSi	2.57	2.41

**Table 2:** Supercell size, K point, atomic number, and GB energy of $\Sigma 3_{Si}$, $\Sigma 3_{C}$, and Σ9 with different C-Si layers.

GB	Layer	L_*x*_ (Å)	L_*y*_ (Å)	L_*z*_ (Å)	K Point	Atoms	$\gamma_{\mathrm{GB}}$ (J/m^2^)
$\Sigma 3_{Si}$	6	6.19	10.72	30.66	4 × 2 × 1	192	1.39
	8	6.20	10.72	40.77	4 × 2 × 1	256	1.39
	10	6.19	10.72	50.89	4 × 2 × 1	320	1.39
$\Sigma 3_{C}$	6	6.19	10.72	30.66	4 × 2 × 1	192	1.39
	8	6.20	10.72	40.77	4 × 2 × 1	256	1.39
	10	6.19	10.72	50.89	4 × 2 × 1	320	1.39
Σ9	15	6.20	9.34	22.11	4 × 3 × 1	120	1.35
	23	6.20	9.32	33.79	4 × 3 × 1	184	1.35
	23 (3 × 1 × 1)	9.29	9.32	33.79	4 × 3 × 1	276	1.35
	31	6.20	9.31	45.47	4 × 3 × 1	248	1.35

## Data Availability

The original contributions presented in this study are included in the article. Further inquiries can be directed to the corresponding author.
